# Curcumin Protects Human Umbilical Vein Endothelial Cells against H_2_O_2_-Induced Cell Injury

**DOI:** 10.1155/2019/3173149

**Published:** 2019-09-02

**Authors:** Jipeng Ouyang, Rong Li, Haiqin Shi, Jianping Zhong

**Affiliations:** Department of Neurology, Shunde Hospital, Southern Medical University, Foshan 528308, Guangdong, China

## Abstract

Migraine is a prevalent neurological disorder which causes a huge economic burden on society. It is thought to be a neurovascular disease with oxidative stress might be involved. Curcumin, one of the major ingredients of turmeric, has potent antioxidative and anti-inflammatory properties, but whether it could be used as a potential treatment for migraine remains to be explored. In the present study, human umbilical vein endothelial cells (HUVECs) were pretreated with various concentrations of curcumin (0 *μ*M, 10 *μ*M, 20 *μ*M, 30 *μ*M, 40 *μ*M, and 50 *μ*M) for 12 h, thereby exposed to H_2_O_2_ (100 *μ*M) for another 12 h. The viability of HUVECs was tested by the CCK-8 assay, and the activities of antioxidant enzymes including superoxide dismutase (SOD) and glutathione (GSH) were also examined. Intracellular reactive oxygen species (ROS) and malondialdehyde (MDA) were assayed to determine H_2_O_2_-induced oxidative stress. In addition, several cell death-related genes (p53, p21, Bax, and Bcl-2) were detected by PCR, and an apoptosis-related protein (caspase3) was evaluated by western blotting. Our results showed that curcumin improved the H_2_O_2_-induced decrease of cell viability and antioxidative enzyme activities and decreased the level of oxidative stress. As a conclusion, curcumin could mitigate H_2_O_2_-induced oxidative stress and cell death in HUVECs and may be a potential therapeutic drug for migraine.

## 1. Introduction

Migraine is a widespread neurological disorder with the typical clinical symptom being recurrent headache [1]. The global prevalence of migraine was reported to be 15%, and 10% will progress to chronic migraine [2]. Migraine-associated functional disability affects patients' work capacity and productivity, thus leading to a huge economic burden on society [3]. Currently, long-term use of migraine drugs may cause some adverse events, such as abuse, addiction, and dependence [4]. As a result, it is of value to conduct more research to promote the understanding of the molecular mechanism of migraine, thus developing novel approaches with improved efficacy and safety.

Curcumin, one of the major ingredients of turmeric, attracted much attention because of its antioxidative, anticarcinogenic, antitumor, and anti-inflammatory properties [5, 6].

A growing body of evidences reports curcumin may have a beneficial antioxidative and neuroprotective potential for neurological diseases such as Alzheimer's disease and Parkinson's disease [7]. As reviewed by Shameemah, 17 studies have revealed the protective effect of curcumin in different cellular models of neurodegenerative disorders [8]. However, to the best of our knowledge, curcumin's treatment potential in migraine has not yet been evaluated.

It is believed that migraine is a neurovascular disorder caused by chronic sensitization of central pain pathways [9]. However, the mechanism of migraine could not be explained by a single theory.

In recent years, oxidative stress has attracted growing interest in the pathogenesis of this disease [10]. For instance, the research conducted by Geyik et al. revealed that oxidative stress marker 8-OHdG was higher in the plasma of migraine patients than that in the control group [11]. Oxidative stress may be caused by a lower activity of certain antioxidant enzymes and a higher activity of oxidant-generating factors [12]. Angiotensin, endothelin-1, and urotensin-2 have been reported to be implicated in this process [13]. Migraines may also be associated with mitochondrial defects, resulting in a much higher metabolic rate. Magnesium, Coenzyme Q10 (CoQ10), and vitamins B2 and B12 have been revealed to have the potential for the treatment of migraine because of their antioxidant abilities [14].

In the present study, we investigated curcumin's effect on H_2_O_2_-induced oxidative stress in HUVECs *in vitro* and aimed to explore the potential use of curcumin in migraine treatment.

## 2. Materials and Methods

### 2.1. Cell Culture

HUVECs were obtained from Cell Bank in the Shanghai Institute for Biological Sciences of the Chinese Academy of Sciences and were cultured in Dulbecco's modified Eagle's Medium (DMEM) medium with 10% fetal bovine serum, 1% penicillin/streptomycin in 37°C, and 5% CO_2_ [15]. All reagents were purchased from Gibco Thermo Fisher Scientific Inc. (MA, USA). HUVECs were treated with various concentrations of curcumin (0 *μ*M, 10 *μ*M, 20 *μ*M, 30 *μ*M, 40 *μ*M, and 50 *μ*M) and H_2_O_2_ (0 *μ*M, 25 *μ*M, 50 *μ*M, 75 *μ*M, and 100 *μ*M, Sigma-Aldrich, St. Louis, MO, USA) 12 h later.

### 2.2. CCK-8 Assay for Cell Viability

The effects of curcumin and H_2_O_2_ on HUVECs viability were detected by the CCK-8 assay. In brief, cells were cultured on a 96-well plate at a density of 1 × 10^4^ per well for 24 h and then administrated with curcumin (0 *μ*M, 10 *μ*M, 20 *μ*M, 30 *μ*M, 40 *μ*M, and 50 *μ*M) for 12 h or with H_2_O_2_ (0 *μ*M, 25 *μ*M, 50 *μ*M, 75 *μ*M, 100 *μ*M) for another 12 h. Then, the HUVECs were incubated at 37°C for 2 h. Thereafter, a multifunctional microplate reader (SpectraMax M5, Sunnyvale, CA, USA) was adopted to read the absorbance values at 450 nm [16].

### 2.3. LDH, GSH, and SOD Assay

In order to estimate the level of oxidative damage, we used a colorimetric assay kit (Beyotime, Nanjing, China) to measure the activity of lactate dehydrogenase (LDH) release [17], superoxide dismutase (SOD), and glutathione (GSH). In brief, HUVECs were seeded in 6-well plates at a density of 1 × 10^5^/well. The HUVECs were then treated for 12 h with various concentrations of curcumin (10 *μ*M, 20 *μ*M, 30 *μ*M, 40 *μ*M, and 50 *μ*M) followed by H_2_O_2_ (25 *μ*M, 50 *μ*M, 75 *μ*M, and 100 *μ*M) for another 12 h.

### 2.4. Analysis of Oxidative Stress

The generation of reactive oxygen species (ROS) was measured using the fluorescent probe 2,7-dichlorofluorescein diacetate (DCFH-DA). The level of intracellular ROS was detected using a BD FACSCalibur Flow Cytometer (Becton, Dickinson and Company, USA). Another indicator of oxidative stress malondialdehyde (MDA) was also detected with commercial kits.

### 2.5. RNA Isolation and Real-Time Quantitative PCR

HUVECs were harvested, and RNA samples were exacted with the TRIzol reagent (Invitrogen, Carlsbad, CA, USA) according to the manufacturer's instructions. The primer sequences used were as follow: p53: 5ʹ-CTTTGAGGTGCGTGTTTGTGC-3ʹ (forward), 5ʹ-TGTTGTTGGGCAGTGCTCG-3ʹ (reverse); p21: 5′-TAGCAGCGGAACAAGGAG-3′ (forward), 5′-AAACGGGAACCAGGACAC-3′ (reverse); Bcl-2: 5′-GTAGTGAATGAACTCTTCCG-3′ (forward), 5′-GTATCCCAGCCGCCGTTCTC-3′ (reverse); Bax: 5′-GACGTGGGCATTTTTCTTAC-3′ (forward), 5′-GTGTCCCGAAGGAGGTTTAT-3′ (reverse); and *ß*-actin: 5′-TGGCACCCAGCACAATGAA-3′ (forward), 5′-CTAAGTCATAGTCCGCCTAG AAGCA-3′ (reverse).

### 2.6. Western Blot

The protein was extracted using the RIPA buffer and measured using the BCA Protein Assay Kit (Beyotime, P0013B, Shanghai, China) according to the instruction. Equal amounts of proteins were separated and transferred to the PVDF membrane (Merk Millipore, Billerica, MA) and incubated with the primary antibodies at 4°C overnight.

This was followed by incubation with the goat anti-rabbit IgG antibody (1 : 10000, Cell Signaling Technology) at room temperature for 2 h. Membranes were scanned by using a chemiluminescent detective system (Amersham Biosciences UK Ltd., Little Chalfont, UK). The primary antibodies anti-cleaved caspase3 (1 : 1000, Abcam, MA, USA) and GAPDH (1 : 1000, Cell Signaling Technology) were used in this study.

### 2.7. Statistical Analysis

SPSS 18.0 for Windows (IBM Corp, Armonk, NY, USA) was used for the statistical analyses. Statistical analyses were performed using one-way analysis of variance (ANOVA) and Student's *t*-test for comparisons between groups. The data were expressed as mean ± SEM, and *p* < 0.05 was regarded as significant differences.

## 3. Results

### 3.1. Cell Viability

**Figure 1:**
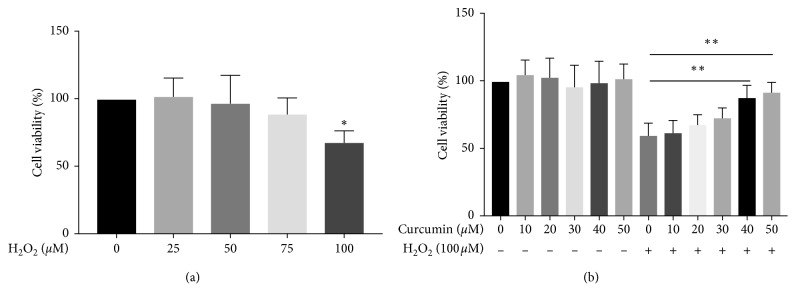
Cell viability of HUVECs treated with curcumin and H_2_O_2_. (a) Treatment with H_2_O_2_ (0 *μ*M, 25 *μ*M, 50 *μ*M, and 75 *μ*M) for 12 h had no effect on cell viability of HUVECs, while H_2_O_2_ (100 *μ*M) decreased cell viability which was tested by the CCK-8 assay. ^*∗*^*p* < 0.05 versus the control group. (b) HUVECs treated with curcumin (0 *μ*M, 10 *μ*M, 20 *μ*M, 30 *μ*M, 40 *μ*M, and 50 *μ*M) for 12 h showed no change of cell vitality. The cell vitality after H_2_O_2_ (100 *μ*M) treatment increased after curcumin (40 *μ*M and 50 *μ*M) administration compared with curcumin (0 *μ*M). ^*∗∗*^*p* < 0.01 (*n* = 3).

To examine the cytotoxicity of curcumin and H_2_O_2_ on HUVECs, cell viability was detected by the CCK-8 assay. As shown in Figure 1, treatment with H_2_O_2_ (0 *μ*M, 25 *μ*M, 50 *μ*M, and 75 *μ*M) for 12 h had no effect on cell viability of HUVECs. However, cell viability of HUVECs decreased when the concentration of H_2_O_2_ increased to 100 *μ*M (*p* < 0.05). Meanwhile, curcumin treatment at the concentrations of 0 *μ*M, 10 *μ*M, 20 *μ*M, 30 *μ*M, 40 *μ*M, and 50 *μ*M showed no cytotoxicity on HUVECs when compared with the control group (*p* > 0.05). Therefore, the concentrations of 0 *μ*M, 10 *μ*M, 20 *μ*M, 30 *μ*M, 40 *μ*M, 50 *μ*M, and 100 *μ*M were chosen for curcumin and H_2_O_2_, respectively, for the subsequent experiments. Furthermore, the decreased cell viability of HUVECs induced by H_2_O_2_ was improved with curcumin treatment (40 *μ*M and 50 *μ*M) (*p* < 0.05), suggesting that curcumin rescued H_2_O_2_-induced cell injury in HUVECs.

### 3.2. The Effect of Curcumin on the Level of LDH Release and GSH and SOD Activity in H_2_O_2_-Exposed HUVECs

**Figure 2:**
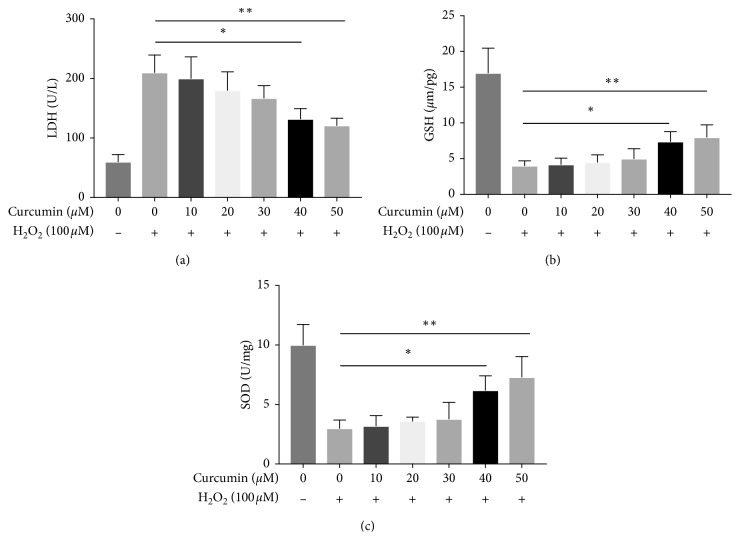
The effect of curcumin on LDH release and GSH and SOD activity in H_2_O_2_-exposed HUVECs. HUVECs were pretreated with curcumin (0 *μ*M, 10 *μ*M, 20 *μ*M, 30 *μ*M, 40 *μ*M, and 50 *μ*M) for 12 h and with H_2_O_2_ (100 *μ*M) treatment for another 12 h. The LDH release and GSH and SOD activity were measured, respectively. ^*∗*^*p* < 0.05 and ^*∗∗*^*p* < 0.01, *n* = 3.

Superoxide dismutase (SOD), glutathione (GSH), and lactate dehydrogenase (LDH) have been widely used as indicators for oxidative injury. As a result, the production of LDH and activities of SOD and GSH were measured to evaluate the effects of curcumin on H_2_O_2_-induced injury in HUVECs. Cells were pretreated with curcumin (0 *μ*M, 10 *μ*M, 20 *μ*M, 30 *μ*M, 40 *μ*M, and 50 *μ*M) for 12 h and with H_2_O_2_ (100 *μ*M) treatment for another 12 h. As shown in Figure 2, H_2_O_2_ administration increased the levels of LDH when compared with the control group (*p* < 0.05). However, the productions of LDH decreased significantly upon treatment with curcumin at the concentration of 40 *μ*M (*p* < 0.05) and 50 *μ*M (*p* < 0.05), even though there was no significant change at the concentrations of 10 *μ*M, 20 *μ*M, and 30 *μ*M (*p* > 0.05). Conversely, the activities of GSH and SOD were enhanced by curcumin (40 *μ*M and 50 *μ*M). Taken together, our result indicated that curcumin showed protective capacity in a dose-dependent manner in H_2_O_2_-induced cell injury in HUVECs.

### 3.3. Curcumin Inhibited H_2_O_2_-Induced Oxidative Stress in HUVECs

**Figure 3:**
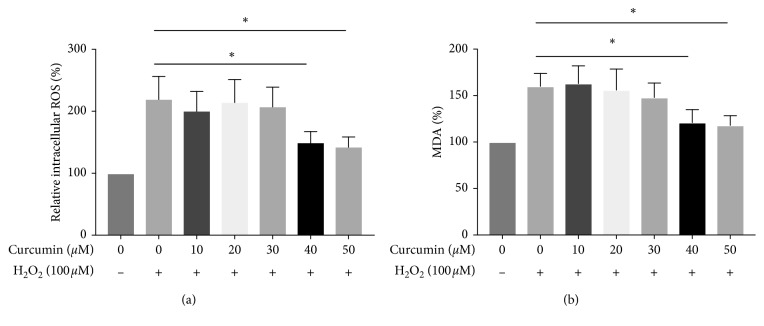
The effect of curcumin on oxidative stress in H_2_O_2_-exposed HUVECs. HUVECs were pretreated with curcumin (0 *μ*M, 10 *μ*M, 20 *μ*M, 30 *μ*M, 40 *μ*M, and 50 *μ*M) for 12 h and with H_2_O_2_ (100 *μ*M) treatment for another 12 h. The DCFH-DA assay was adopted to detect endogenous ROS, and the commercial MDA kit was used to examine the MDA level. ^*∗*^*p* < 0.05, *n* = 3.

In order to test whether curcumin could influence oxidative stress in HUVECs, we examined the generation of the intracellular ROS and MDA level. As is shown in Figure 3(a), intracellular ROS was increased in HUVECs under H_2_O_2_ treatment (*p* < 0.05). In addition, H_2_O_2_ treatment also caused a significant induction of another oxidative stress marker, malondialdehyde (MDA) (Figure 3(b)) (*p* < 0.05). Notably, a significant reduction of both the intracellular ROS and MDA level was observed with curcumin pretreatment (40 *μ*M and 50 *μ*M) for 12 h. In contrast, the curcumin concentration of 10 *μ*M, 20 *μ*M, and 30 *μ*M had no effect on in H_2_O_2_-induced HUVECs in terms of either intracellular ROS or MDA level (*p* > 0.05). Our data suggested that the effect of H_2_O_2_ on the intracellular ROS and MDA levels in HUVECs could be blocked by curcumin at a certain concentration.

### 3.4. The Effect of Curcumin on Cell Death-Related Genes in H_2_O_2_-Exposed HUVECs

**Figure 4:**
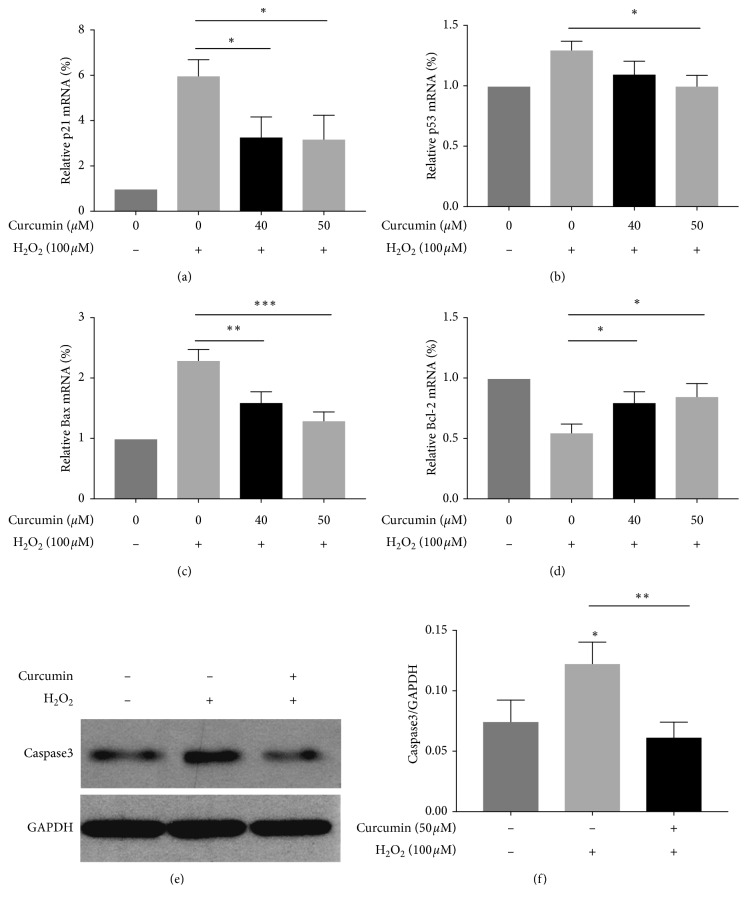
The effect of curcumin on cell death-related genes in H_2_O_2_-exposed HUVECs. HUVECs were pretreated with curcumin (40 *μ*M and 50 *μ*M) for 12 h and with H_2_O_2_ (100 *μ*M) treatment for another 12 h. The mRNA level of cell death-related genes including p53, p21, Bax, and Bcl-2 was detected by PCR. The expression of apoptosis protein caspase3 was evaluated by western blot. ^*∗*^*p* < 0.05 and ^*∗∗*^*p* < 0.01, *n* = 3.

In order to elucidate the effect of curcumin on cell apoptosis, the expression of cell apoptosis-related genes, including p53, p21, Bax, and Bcl-2, was examined by PCR. The result showed that H_2_O_2_ induced a significant increase in apoptosis-related genes (p53, p21, and Bax) and a significant decrease of antiapoptotic gene (Bcl-2) (Figure 4) (*p* > 0.05). Notably, after curcumin (40 *μ*M, 50 *μ*M) treatment, these effects were partly reversed. Moreover, the expression of apoptosis-related protein was evaluated by western blot. As shown in Figure 4(e), the caspase3 level was significantly reduced by curcumin treatment (50 *μ*M). Therefore, we suggested that curcumin had a protective effect on HUVECs against oxidative stress.

## 4. Discussion

In the present research, our results showed that curcumin could mitigate H_2_O_2_-induced decrease of cell vitalities and antioxidative enzyme activities and decrease the level of oxidative stress in HUVECs. Curcumin, an active compound isolated from turmeric, has been demonstrated to have protective effect on neurological disorders [18]. However, as we know, there is still no published paper reported on the role of curcumin in model of migraine.

The safety of curcumin has been well proved [19]. Our result also showed that curcumin at the concentration of up to 50 *μ*M had no effect on the cell viabilities of HUVECs. Notably, the concentration of 40 *μ*M and 50 *μ*M showed a property to inhibit H_2_O_2_-induced oxidative stress (Figure 1). Furthermore, our results also showed that curcumin suppressed H_2_O_2_-induced induction of p53, p21, and Bax while enhanced the expression of antiapoptotic gene Bcl-2 (Figure 4).

The oxidative stress is mostly caused by the imbalance of ROS production and the clearance system [20]. It has been reported that curcumin rescued 6-OHDA-induced reduction of the antioxidant enzymes GSH, GPx, GR, and SOD [21]. Harish et al. exposed curcumin-pretreated N27 cells to buthionine sulfoximine and observed the upregulation of GSH and glutathione S-transferase [22]. The effect on GSH levels was also observed in a lipopolysaccharide-induced mouse model with curcumin treatment for seven days [23]. Consistently, we also observed an upregulation of SOD and GSH levels, suggesting that curcumin could regulate oxidative homeostasis in HUVECs by stimulating the activities of antioxidant enzymes.

Increasing evidences have reported the role of oxidative stress in migraine [11]. As shown in Figure 3, curcumin could ameliorate H_2_O_2_-mediated oxidative stress by reducing ROS and MDA levels. This is similar to the experiment performed by Cui et al. who applied rotenone to induce oxidative stress in the dopaminergic neuron [24]. In addition, another study investigated the antioxidant properties of curcumin also used H_2_O_2_ to stimulate oxidative stress. The authors suggested that curcumin was capable of improving both DNA damage and cell apoptosis in PC12, which is an established cell model of PD [25]. However, the effect of curcumin against oxidative damage depends on the cell line. van Meeteren et al. observed poor efficacy of curcumin in H_2_O_2_-stimulated oligodendrocytes and macrophages cells [26]. In the present study, we firstly showed that curcumin had the protective effect on H_2_O_2_-induced oxidized proteins and lipid radicals in HUVECs.

The molecular mechanism of the protective effect of curcumin on HUVECs under oxidative stress still remains to be explored. By exploring the antioxidant effect of curcumin in amyloid-*β* exposed SH-SY5Y and IMR-32 cells, Sarkar et al. proposed that it might be associated with the activation of antioxidant element and nuclear factor erythroid 2-related factor 2 (Nrf2) pathways [27]. In another study, the reduction of oxidative stress by curcumin only occurred in cells with mutant *α*-synuclein, suggesting that the oxidative stressor might be essential for the antioxidant effect of curcumin [18]. Another study with a model PC12 cells showed that mitogen-activated protein kinases (MAPKs) and serine/threonine protein kinase (Akt) pathways might play a vital role in the antioxidant effect of curcumin [25]. However, whether the mechanism is implicated in HUVECs is unclear.

In the present experiment, DMSO was adopted as the solvent for curcumin. Previous published papers reported that DMSO might change the conformation of curcumin [28]. However, according to the published paper, the working concentration of DMSO we used in this research (0.1%) is not likely to cause effect on curcumin [29]. With high lipophilicity, curcumin is free to pass through cellular membranes, which might be the basis of the effect in HUVECs [30]. However, the potential application of curcumin in clinical trials is largely hindered by its poor bioavailability [31]. Its low intestinal absorption, poor structural stability, and rapid metabolism, especially the poor permeability across the blood-brain barrier, are major obstacles for the use of curcumin as a therapeutic agent for migraine [19]. As a result, various drug delivery approaches have been used to increase curcumin's bioavailability [8]. It has been demonstrated that structural alteration and bioconjugates enhanced the curcumin's protective property against oxidative stress [23]. In addition, lots of researches showed that nanocarriers including liposomes, isomerization, polymeric nanoparticles, and polymeric micelles rendered a larger effect size for curcumin [32, 33]. For instance, Khalil et al. illustrated that the PLGA-PEG nanoparticles increased the bioavailability of curcumin by up to 55.4-fold by decreasing the degradation [34]. With the improvement of nanotechnology, curcumin would be more likely to be used to treat migraine.

To the best of our knowledge, there is no widely accepted *in vitro* model for migraine. Both C2C12 myoblasts and meningeal mast cells [35] have been used as model for migraine research [36]. Harriott et al. also suggested the spreading depression model to be used as a preclinical model of migraine [37]. Since migraine is a vascular disorder which was thought to be related to the trigeminovascular pathway, we used HUVECs as a model to study the mechanism (oxidative stress) that may be associated with migraine. There are limitations to the approach of using HUVECs as a migraine model. The model we used is just one component part of the complex heterogeneous pathogenesis of migraine; however, we think it could be helpful for the examination of alterations in vascular dysfunction. The effect of curcumin on migraine remained to be confirmed in an *in vivo* experiment and clinical trial.

## 5. Conclusion

In the present study, we report the effect of curcumin on H_2_O_2_-induced oxidative stress in HUVECs, which might be a potential therapy for migraine.

## Data Availability

The data used to support the findings of this study are available from the corresponding author upon request.
